# Resource management in fisheries under different types of externalities in a two-country general equilibrium model of international trade

**DOI:** 10.1016/j.heliyon.2023.e18362

**Published:** 2023-07-17

**Authors:** Gökhan Güven

**Affiliations:** Department of Economics, Faculty of Political Sciences, Sakarya University, 54050, Serdivan, Sakarya, Turkey

**Keywords:** International trade, Immiserizing resource management, Production externalities, Fishery resources

## Abstract

Open access resource problems and harmful pollutants from manufacturing activities are common in resource management practices. Nevertheless, their implications have only been studied in different and separate frameworks that are not covered within the same structure. Previous studies suggest that resource management enforced by one country can increase welfare levels and rebuild resource conservation, compared to the case where no country imposes resource management policies. However, in real-life examples, the harvesting and manufacturing industries exert simultaneous pressure on fishery resource stocks, thereby changing the nature of the supply curve of renewable resources. This study investigates the effects of trade liberalization under unilateral resource management regimes in a two-country, two-sector model, in which both production sectors can detrimentally affect renewable natural resources by generating two interacting environmental burdens: excessive harvesting and industrial pollution. It is demonstrated that unilateral resource management applied by a country in which the resource-good sector is relatively less damaging to fishery stocks is welfare-reducing for both countries compared to the situation where neither manages its resource sector. This result is identified as “immiserizing resource management.” Notably, however, unilateral resource management by one country in which the resource-good sector has a more significant negative impact than the manufacturing industry can benefit both trading partners in welfare terms; this is referred to as “improving resource management.” Policymakers in international organizations should consider the relative dominance of externalities in the presence of weak property rights before requiring resource management as a condition for participating in international trade.

## 1 Introduction

It is commonly argued that renewable natural resources (such as fisheries) tend to be characterized by detrimental externalities stemming from ill-defined property rights or a lack of adequate environmental control policies [1,2]. According to existing literature, the socially optimal use of fishery resources differs from the private use of such resources because of poorly delineated and illiberal defined controlling and monitoring regimes, which can explain to a certain extent why natural resources are prone to depletion, especially in developing countries [3]. In line with this argument, many economists believe that fishery resources are identified by their open-access characteristics, in which input factors are believed to be allocated to the fishery sector higher than their optimal level, which is supposed to be found when property rights are appropriately enforced [4].

Furthermore, trade liberalization is usually expected to cause overfishing and, ultimately, resource extinction in some areas because of the nature of the fishery resource being considered a market failure. The impact of openness on the degree of excessive consumption of fishery resources depends on the relative level of management regime [5]. Laxer environmental regulations on fisheries lead to a variety of economic fluctuations in post-trade production patterns and unsustainability in fishery harvesting [6]. The critical point in mitigating the associated loss with incomplete property rights in the presence of open-access problems exacerbated by trade openness is to enforce completely designed property rights, which would generate genuine information about the costs and benefits of using common-pool resources for all economic agents in the market. However, rights-based solutions and arrangements for resource extraction problems are complicated and costly [7]. For this reason, policymakers seek feasible and efficient alternative approaches to address open-access problems, such as implementing resource management regimes to regulate at least one type of externality that deteriorates natural resources.^1^ The research gap dealt with in the present study is that it has yet to be theoretically analyzed whether or not unilateral resource management is effective and welfare-improving when developing countries with two different and interrelated environmental distortions open up to trade.

The present model uses a fishery resource model with two countries (domestic and foreign) and two goods (resource and manufacturing goods) in the presence of two detrimental externalities: excessive harvesting and industrial pollution, to investigate the effect of resource management regimes on post-trade production patterns, welfare, and resource conservation terms. In this model, each country enforces different technology standards in the harvesting industry, resulting in differences in autarkic steady-state resource stocks and providing the motivation behind the trade between both countries. The heterogeneity in production efficiency in the resource-intensive sector in both countries is assumed to determine the environmental and welfare consequences of trade. By incorporating these assumptions, the model obtains three main results.

This study differs from existing literature in two ways. Instead of analyzing the impacts of unilateral resource management regimes on resource stocks by considering two different externalities separately, I investigate the consequences of unilateral resource management within the framework of a model in which externalities exist simultaneously, but to different degrees.^2^ Second, much of the existing literature does not sufficiently consider the change in resource stocks over time, implying that resource dynamics are not considered. However, the present model focuses on the evolution of natural resources and emphasizes the importance of the magnitude of adverse effects in determining the direction of resource stock preservation.

First, I find a novel result: in the presence of weak and incomplete property rights, the implications of resource management regimes depend on the relative dominance of economic activities in terms of generating environmental burdens on resources. At first glance, even if their trading partners do not implement a resource management policy, they are still interested in enforcing it unilaterally. However, an optimal unilateral management policy requires that the country take this action, in which the resource extraction problem dominates the industrial pollution externality owing to its detrimental effects. By doing this, the model provides a win-win scenario in which fishery management is put into effect by a country where harvesting is a more resource-depleting activity than is manufacturing. This happens because resource management in such a country induces labor to move from the less-efficient sector to the more productive industry, which implies higher welfare and resource protection. This case is referred to as the “improving resource management” policy, which is in accordance with the standard trade theory.

Second, the welfare analysis indicates that under the assumption that the relative detrimental strength of industrial pollution dominates open-access excessive harvesting, the worst scenario occurs: free trade leads to environmental degradation in both countries, thus harming utility results. This case is of particular attention because it shows that in the absence of appropriate management of environmentally more damaging industries, resource management enforced in only one country could result in welfare loss in each country. Here, it is essential to note that trade can result in a more distorting industry expansion at the expense of contracting more productive economic activity in each country when unilateral resource management is enforced by the economy, where manufacturing inflicts relatively more depletion on fishery resources. This scenario is called “immiserizing resource management.”

Finally, and critically, all these results generate significant political implications; policymakers should pay attention to the relative relationship between externalities and the prioritization of economic activity that is more detrimental to the resource stock when considering resource management policy. This implies that policies should be situation-specific. Therefore, the requirements of international organizations for countries to practice resource management as part of foreign trade may have unintended welfare-reducing effects. Consequently, the present model suggests that organizations such as the WTO should avoid generalizations about the benefits of free trade and its impacts on resource endowments in the presence of weak property rights. This is key to understanding the role of resource management in international trade and is crucial for ensuring that resources are managed effectively and sustainably.

This study builds on the literature that investigates the effects of international trade on natural resources [[8], [9], [10], [11], [12], [13], [14], [15], [16], [17], [18], [19], [20]]. [8] analyzes trade relations between developing countries and shows that the exploitation of natural resources is exacerbated by emerging countries' increased access to international markets [9]. merit attention because they state that natural resources tend to be generally over-depleted, and trade openness incentivizes environmental deterioration [10,11]. find that in the presence of open-access renewable resources, trade liberalization may harm at least one trading partner's welfare and claim that resource management can generate welfare-improving terms in both trading partners [14]. These studies suggest that if a country cannot manage its extraction rates, it can experience post-trade welfare loss and resource depletion.

[15] also constructs a model in which the carrying capacity of the resource is endogenously determined [16]. analyzed the importance of tax regulations in the presence of shared maritime activities, implying that other countries' tax strategies on resources affect common resource dynamics [17]. scrutinize theoretical differences in environmental regulation regimes and conclude that stricter management policies are able to diminish productivity in the resource-intensive sector if countries share a common resource stock [19]. study the effects of free trade on utility terms by decomposing welfare effects into different parts and resulting in the detrimental impact of trade openness not being short-sighted and its impact getting more prominent over time [18]. incorporated two kinds of environmental burdens (overharvesting and pollution) into the analytical model to examine post-trade resource protection and welfare consequences from trade [20]. can be considered an extension of the previous paper, and they develop a model in which the relative damage inflicted by different economic activities categorizes the countries into two extreme points. They find that when the two countries are classified into different types, a pessimistic scenario may occur, resulting in trade harming both trading partners.

One may have realized that this model's structure is similar to that of [18,20]. This is no surprise because the present model also analyzes the simultaneous existence of two interrelated adverse economic activities on natural resources, as in the other two studies. Their analysis was confined to the framework and no resource management was assumed. The present study, however, provides a clear explanation of how a resource management regime could change welfare with respect to relatively harmful impacts, which has not been covered in these studies.

A significant body of the literature examines resource management in the presence of open-access resources [21]. considered unilateral resource management and showed that trade-induced losses can emerge in both countries. The mechanism inducing this consequence is distinct from the findings of this model in two ways. First, the environmental distortion in their model is only related to the open-access problem, which stems from the enforcement of imperfect property rights. By contrast, this model considers two sources that stimulate environmental failure. Second, in their model, resource stock dynamics do not play a crucial role in welfare changes. In this model, stock evolution is considered the driving force that creates utility differences in the pre- and post-trade results.

[22] proposes a model in which endogenous resource management is assumed to be enforced at a cost and finds that trade openness with appropriate resource management can lead to different welfare consequences, implying that resource management does not always cause effective management [23]. differentiates countries in terms of resource abundance. By creating heterogeneity in countries, they analyzed the introduction of resource management standards in trading countries. However, he only examined the relationship between resource-abundant countries and resource management policies in ascertaining post-trade production specialization and welfare patterns. In contrast, the present model extends this discussion by incorporating the conditions under which resource-scarce countries could be affected by resource management strategies by changing the relative impact of detrimental spillovers of economic activities.

[24] empirically study the factors that affect the social planner's decision on the efficiency of fishery resource management policy [25]. examine the strategic interaction between trade and the environment under multilateral resource management decisions enforced by both countries. Assuming a shared resource assumption, their analysis concentrated on particular cases in which both trading partners had to have a common renewable resource stock. However, this study assumes that each country is endowed with its own resource stocks, allowing us to investigate how unilateral resource management practices impact resource conservation and, consequently, welfare terms within the country [26]. presented the relationship between productivity enhancements and optimal resource management in fisheries and found that improved technology standards require a more stringent management regime and increased productivity in the harvesting sector.

The key novelty of this study is the ability to understand the motivations that determine post-trade welfare results in unilateral resource management enforced by trading countries where relative damage caused by different economic activities exists. No theoretical model exists that deals with welfare gains/losses and resource preservation under the management of resource stocks defined within national borders when both externalities exist. Depending on the more sophisticated setting of interrelated externalities, this study shows how an opening up of trade affects the utility levels of economies by enforcing resource management practices.

This paper also contributes to the literature by presenting analytical results that are more consistent with real-life examples. This is because previous studies have analyzed the effectiveness of resource management strategies in a framework in which only one externality impacts resource dynamics over time [21,27,28]. However, renewable resource stocks (e.g., open-access fishing areas) are generally located near polluter-producing firms. Thus, the simultaneous existence of two types of pressures must be considered, as in this model, which is the main originality of this study.

This study examines the effects of resource management enforced by only one country in an economic framework in which two different types of interrelated externalities damage resources simultaneously, which certainly alters the nature of production possibilities frontiers in a two-country, two-good general equilibrium model. In other words, the primary research question addressed in this study is whether there is any possibility in which both countries can experience welfare-enhancing benefits and improved resource conservation, given that only one country manages its fishery resource sector. The motivation of this study is to better understand how unilateral resource management policy behaves under different environmental pressures and to determine the conditions under which it can enrich or impoverish trading partners. To the best of my knowledge, no previous analytical study has examined the relationship between resource management and international trade in the presence of different externalities.

The remainder of this paper is organized as follows. The next section provides the basic structure of the proposed model. Section 3 incorporates the open economy case into the model characterizing the unilateral resource management policy and allows the examination of post-trade conservation and welfare results. Section 4 investigates the results of resource management enforced by only one trading partner country, focusing on how these results compare with free trade under the condition that both countries are subject to different levels of relative damage. The last section discusses policy-relevant questions and explores their political implications.

## 2 The basic model

The method in this model restricts particular attention to comparative steady-state analysis with diversified equilibrium. First, autarkic steady-state points are identified. What follows is that economies are open to trade, and introducing a resource management regime enforced by only one trading partner is assumed. Finally, this study compares the impacts of resource management practices on pre-trade, post-trade welfare, and environmental preservation results.

The present model assumes that the fish stock (S) grows at the natural growth rate of the fishery resource, denoted by G, which satisfies the conditions G(K)=0, and $G^{\prime }\left(K\right)<0$ for a non-negative value of K, where K represents the maximum carrying capacity.^3^ The natural growth rate is a closed functional form of available fishery stocks. For a given stock size, the Schaefer-type production function of renewable resource harvest is given by Eq. (1),^4^

(1) $$H_{S}=qA\left(S\right)L_{H}$$

in which q refers to the parameter measuring the technology level for harvesting (H), $L_{H}$ is the labor for harvesting, and A(.) is a positively correlated function with renewable resources, satisfying that $A^{\prime }\left(S\right)>0$ (henceforward, subscripts “S” and ″D″ denote “production” and “consumption,” respectively). The other industry, manufacturing (M), produces an average of some other goods with constant returns to scale production technology, as shown in Eq. (2).

(2) $$M_{S}=L_{M}$$

where $L_{M}$ is the manufacturing labor. The manufacturing industry produces harmful pollution, Z, on fishery stocks at a positive and constant pollution intensity parameter (γ) throughout the production process, identified as $Z=\gamma M=\gamma L_{M}$. Industrial pollution is another externality that generates additional environmental burdens on fishery resource stocks.^5^

Before proceeding to obtain a steady-state equilibrium, we must identify more precisely what we mean by the relative dominance of different kinds of externalities in terms of harmful activities. The detrimental impact on resource stocks per unit of labor in the fish harvesting industry is qA(S), followed by γ in the manufacturing industry. It is crucial to determine which activity dominates over the other, as their relative harmfulness determines the nature of the production possibilities frontier in the autarkic economy and the production/trade patterns when the economy opens up to trade. The following definition summarizes the associated discussion:

Definition 1 The fish harvest sector is assumed to be more resource-depleting than the manufacturing sector, as the pollution intensity parameter is lower than the threshold level; [γ<qA(S)]. Conversely, the manufacturing sector becomes more environmentally damaging when the parameter is sufficiently high [γ>qA(S)].^6^

The model divides economies into two types, depending on whether the open-access problem has more negative effects than industrial pollution, which will be discussed in more detail in subsequent sections.

In this model, open-access fishery stocks are subjected to two interrelated problems: excessive harvesting and industrial pollution.^7^ In the steady state, the natural growth rate of the fishery resource is equal to the production of the resource-good and industrial pollution, meaning that G[S(t)]=[H(t)+Z], and then

(3) $$\frac{dS}{dt}=Ś=G\left(S\right)-\left[H\left(t\right)+Z\right]=G\left(S\right)-T=0,and\;where\;T=\left[H\left(t\right)+Z\right]$$

where T describes the total level of externality that negatively affects the steady-state resource stocks. Given the labor allocation, β, the following expression is obtained based on equation (3), G(S)=L[qA(S)β+γ(1−β)]. The solution(s) to this steady-state fishery stock expression is identified by $S_{\infty }\left(\beta \right)$, which requires satisfying the following stability condition:

(4) $$G^{\prime }\left(S\right)<A^{\prime }\left(S\right)L\left[q\beta \right]$$

This stability condition guarantees that the natural growth rate of fishery resources intersects the total detrimental burdens from economic activities on resources, T, from above. By taking the total differential of G(S)=L[qA(S)β+γ(1−β)] to β, it generates the following expression (see Refs. [18,20]):

(5) $$\frac{dS_{\infty }\left(\beta \right)}{d\beta }=S_{\infty }^{\prime }\left(\beta \right)=\frac{L\left[qA\left(S_{\infty }\left(\beta \right)\right)-\gamma \right]}{G^{\prime }S_{\infty }\left(\beta \right)-A^{\prime }\left(S_{\infty }\left(\beta \right)\right)L\left[q\beta \right]}$$

Based on the stability requirements mentioned in Eq. (4), denominator of Eq. (5) must be negative, which means that the opposite sign of the numerator determines the sign of Eq. (5). Here, the model assumes that the production technology in the renewable resource harvest sector is different in both countries, as follows: $q<q^{*}$.^8^ This implies that the foreign country produces its resource-good with higher efficiency, as compared to that in the domestic country. Based on this assumption, it is reasonable to assume that in a country with lower technological efficiency, the pollution parameter may be more damaging to resource stock.

Therefore, recalling Definition 1, when manufacturing activity is more harmful to fishery stocks, γ>qA(S), the model assumes the economy as in the domestic country. On the other hand, when the excessive harvesting problem dominates industrial pollution in its adverse impacts on fishery resources, $\gamma <q^{*}A\left(S\right)$, the model assumes the economy as in the foreign country. Differences in country types ensure that a marginal increase in labor allocation, $\beta \;\left[\beta^{*}\right]$, brings about the rebuilding (degradation) of the steady-state fish stock, which consequently implies that $S_{\infty }^{\prime }\left(\beta \right)>0$ ${[S}_{\infty }^{\prime }\left(\beta^{*}\right)<0]$ for the domestic (foreign) country, respectively, and^9^

Lemma 1 In a domestic (foreign) country with a pollution intensity parameter above (below) its critical value, $S_{\infty }^{\prime }\left(\beta \right)>0$ $\left[S_{\infty }^{\prime }\left(\beta^{*}\right)<0\right]$ holds for all $\beta ,\beta^{*}є\;\left[0,1\right]$.^10^

*The full employment condition*, $L=L_{H}+L_{M}$, *and*$L_{M}=M_{S}$, *then*$M_{S}=L-\frac{H_{S}}{qA\left(S\right)}$. *In the steady state*, $H_{S}=G\left(S\right)-\gamma \left(1-\beta \right)L$, *and thus*, $M_{S}=L-\frac{G\left(S\right)-\gamma \left(1-\beta \right)L}{qA\left(S\right)}$. *The relative supply curve at the steady state is defined as follows*:

(6) $$\frac{H_{S}}{M_{S}}=\frac{G\left(S\right)-\gamma \left(1-\beta \right)L}{L-\frac{G\left(S\right)-\gamma \left(1-\beta \right)L}{qA\left(S\right)}}$$

Eq. (6) *is a helpful tool for determining the sign of the relative price of a harvesting good in terms of the relative supply curve*, *yielding the following proposition*:^11^

Proposition 1 *The domestic* (*foreign*) *country’s relative price of the harvesting good to the manufacturing good* $\left(p_{H}\right)$ *is downward* (*upward*) *sloping in terms of the relative supply of harvesting to manufacturing* $\left(H_{S}/M_{S}\right)$. *This implies that* $\frac{dp}{d\left[H/M\right]}<0$ *and* $\frac{dp^{*}}{d{\left[H/M\right]}^{*}}>0$ *for domestic and foreign countries*, *respectively*.

***Proof***. *It is assumed manufactured goods in each country are produced under perfectly competitive market conditions*. *Furthermore*, *the value of the marginal product of labor in the*M*industry must be equal to the wage rate*(w), *implying that*$p_{M}\left(\frac{\partial M_{S}}{\partial L_{M}}\right)=1$*for both countries*. *Moreover*, *the profit-maximization condition in the fish harvest sector implies that*$p_{H}=\frac{w}{qA\left(S\right)}=\frac{1}{qA\left(S\right)}$. *In the steady state*, $S_{\infty }\left(\beta \right)\text{,}$*the relative price is defined as follows*: $p=\frac{1}{qA\left[S_{\infty }\left(\beta \right)\right]}$, *and then*$\frac{dp}{d\left(H/M\right)}=\frac{dp}{d\beta }\frac{d\beta }{d\left(H/M\right)}$*where*M=(1−β)L.*Taking the differential provides the following expression*$\frac{dp}{d\beta }={-\left[qA(S_{\infty }\left(\beta \right)\right]}^{-2}\left[qA^{\prime }(S_{\infty }\left(\beta \right)\right]S_{\infty }^{\prime }\left(\beta \right)$*in which*${-\left[qA(S_{\infty }\left(\beta \right)\right]}^{-2}<0$*and*$A^{\prime }\left(S\right)>0$. *Furthermore*, $H/M=\left(qA\left(S\right)\right)\frac{\beta }{\left(1-\beta \right)}$*indicates that*$\frac{d\beta }{d\left(M\right)}>0$. *Considering these two differentials together and depending on*Lemma 1, $S_{\infty }^{\prime }\left(\beta \right)>0\;\left[S_{\infty }^{\prime }\left(\beta^{*}\right)<0\right]$, *and then it follows that*$\frac{dp}{d\left(H/M\right)}<0\;\left[\frac{dp^{*}}{d{\left[H/M\right]}^{*}}>0\right]$*for the domestic* (*foreign*) *country*.

An interesting feature of this relative supply curve is that it is upward-sloping in the domestic country. This is because that manufacturing is a more resource-depleting economic activity in the domestic country, resulting in one unit of labor shifting from H to M, leading to the degradation of fish resources at the steady-state equilibrium. From the condition $=\frac{1}{qA\left[S_{\infty }\left(\beta \right)\right]}$ , it follows that the relative price of the harvesting good declines. The same reasoning can be applied to the foreign country, except that an extra worker transferred from $H^{*}$ to $M^{*}$ will have a steady-state resource-improving impact because harvesting is more harmful to fishery resources. Therefore, the relative price of the resource-good in the foreign country increases.

## 3 International trade between two countries under the open-access problem

The model consists of two countries, domestic and foreign, distinguished by whether the pollution parameter is above the threshold value. As in other trade models with two externalities, the world price after trade lies between the autarky prices. In this situation, if both countries are differentiated in terms of the relative dominance of economic activities (e.g., the open access problem dominates industrial pollution in the domestic country, and vice versa for the foreign country), the long-run welfare implications can be summarized as follows:

i) When both countries export their relatively more “environmentally friendly” goods to one another (implying that the domestic country exports the H-good and, the foreign country exports the M-good), either of them experiences welfare gains from opening up to trade.
ii) When domestic and foreign countries export their relatively “dirtier” goods to one another (implying that the domestic country exports the M-good and the foreign country exports the H-good), both countries encounter welfare-reducing consequences from trade liberalization.

This is because international trade leads to labor shifting to a more resource-depleting sector in both countries, resulting in a deterioration of fishery resource stocks and lower efficiency in the fish harvesting sector in both countries. These results are also indicated by each country's utility function as follows:

The direct utility function for both countries can be assumed in Cobb-Douglas form for a representative consumer, implying that $u={\left[H_{D}\right]}^{\beta }{\left[M_{D}\right]}^{1-\beta }$ where $H_{D}=\frac{\beta Lw}{P_{H}}$ , $M_{D}=\left(1-\beta \right)Lw$ and $p_{H}=\frac{1}{qA\left(S\right)}$. Substituting these equations into the direct utility function and applying a monotonic transformation, it follows that at steady-state equilibrium, $V^{open}=lnu=\Lambda +lnL+lnw+\beta \;\ln\left[qA\left(S_{\infty }\left(\beta \right)\right)\right]$ where Λ=βlnβ+(1−β)ln⁡(1−β). The real income in the two countries with open-access resource sectors is wL=L, and $V^{open}=\Lambda +lnL+lnw+\beta \;\ln\left[qA\left(S_{\infty }\left(\beta \right)\right)\right]$. The last expression reveals a positive relationship between utility and fishery resources.^12^

In this situation, trade liberalization does not lead to welfare gains traditionally attributed to it. The observation of welfare losses in both countries is not surprising to environmental economists, as the existing literature shows that free trade increases the world demand for products produced at relatively lower costs, resulting in a decline in fishery resource stocks in both countries.

The most obvious solution to reducing welfare losses in free trade is to encourage both countries to bolster their property rights and establish an appropriate legal framework [21,29]. According to existing literature, well-defined property rights and appropriate economic measures can enhance environmental quality and bolster long-term environmental protection [1,30]. To achieve this, countries can implement resource management and/or pollution control measures [31,32].^13^ However, there is an ongoing debate between natural resource management and pollution control mechanisms over their relative efficiency and effectiveness in terms of rebuilding resource stocks and improving comparative advantage [33].

Furthermore, it is expensive and difficult to implement resource management policies and pollution control regulations at the same time due to the disagreement among stakeholders regarding the significance of sustainable resource utilization and the potential economic and political conflicts of interest involved. Pollution-intensive producers are more likely to oppose pollution control measures, as these regulations can increase their production costs and reduce their competitiveness [34].

Additionally, an increasing trend in pollution during industrialization increases the difficulty of implementing pollution-control regimes [35,36]. Therefore, this model introduces a resource management strategy rather than pollution control, without identifying any particular management strategies. It is assumed that maximizing resource rents for resource extraction is sufficiently valuable. In light of these discussions, the central question of this study is whether both countries can obtain welfare gains if only one country manages its fish harvest sector, regardless of whether the open-access problem is a significant depleting activity in the regulating country? It must be noted that while the resource management policy serves as the central policy to preserve fisher resource stocks (due to the relative dominance of harvesting in its negative impacts) in the foreign country, this policy would direct labor to more-damaging sectors in the domestic country.

Lastly, renewable resource management can be regarded as a dynamic allocation problem [37]. One approach would be to implement quota management. However [38], proved that quota measures on resources were inadequate to satisfy environmental and economic targets simultaneously. Another management approach is a community-based co-management strategy in which both countries’ fisheries and stakeholders participate in management decisions [39]. The combination of a well-designed bureaucratic state and independent private economic agents is a significant component of effective implementation [40]. The exclusive access rights to local fishing grounds by all trading partners can be viewed as another management system. However [41], claim that ill-defined property rights and laxer environmental measures in developing and integrated economies complicate such collaborative action between trading partners. In summary, it is logical to examine a one-sided resource management strategy in the presence of trade between developing nations.

### 3.1 Resource management by the foreign country

For simplicity, the model neglects the analysis of dynamic resource allocation and concentrates on issues related to the static externality stemming from ill-defined property rights. Under open access, resource rents associated with extraction are dissipated in the long term because there is no regime of property ownership over fish resources to prevent other economic agents from entering the market to such an extent that rents are canceled out. Therefore, the resource extraction problem is considered an externality that needs to be corrected through resource management. The rest of the model is based on comparative steady-state equilibrium analysis.

**Fig. 1:**
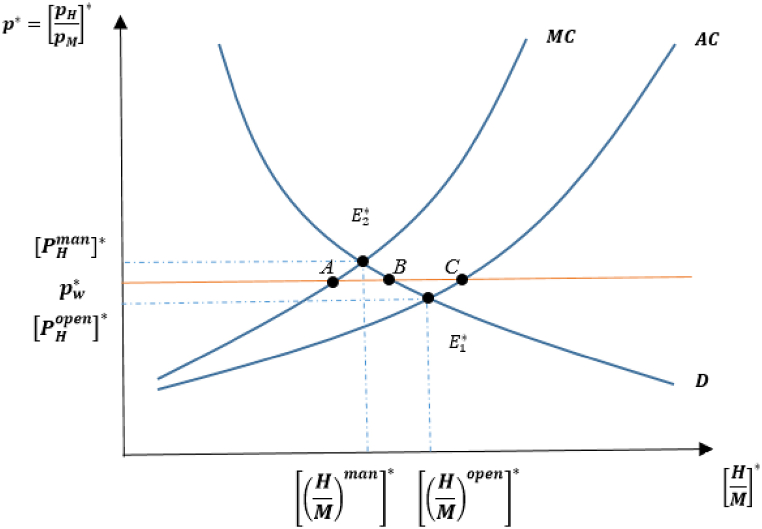
Open access and managed equilibria in autarky for foreign country.

The model initially focuses on the case in which only a foreign country, [H(t)>Z], manages its fishery resource sector.^14^ Assuming that trade occurs between domestic and foreign countries with identical utility and the natural rate of resource growth, the reason for trade emergence is that each country has a differentiated fish harvest technology, resulting in different levels of autarkic steady-state equilibriums (and consequently different levels of the steady-state relative price of the resource good). This case is illustrated in Fig. 1 using the relative average cost (AC), marginal cost (MC), and demand (D) curves for the foreign country.^15^

The proposition reveals that the relative price of the H-good to the M-good is upward-sloping concerning the relative supply curve in the foreign country, as depicted in Fig. 1. Resource management leads to labor migration from the fish harvest industry to the manufacturing sector, implying that more labor is left over to produce M-goods. Depending on the decline in $L_{H}^{*}$ in the managed country, fish resource stocks remain higher in the open-access, compared than in the managed country. Therefore, the relative average cost curve lies outside the marginal cost at the autarkic steady-state equilibrium, as shown by ${\left[{\left(\frac{H}{M}\right)}^{man}\right]}^{*}<{\left[{\left(\frac{H}{M}\right)}^{open}\right]}^{*}$ in Fig. 1.

In the foreign country, managing its fishery harvest sector equilibrium is indicated by $E_{2}$ in autarky, in which demand intersects the marginal cost. In the open-access case of a foreign, an autarkic equilibrium is established at $E_{1}^{*}$.^16^ Assuming that trade takes place between these types of countries, the free-trade world price takes a value between the autarky prices, denoted by $p_{w}^{*}$. Trade liberalization causes welfare gains (losses) for the managed (open access) country, compared to their autarky levels. The intuition behind this result is as follows: The “managed” country, whose autarky price is ${\left[p_{H}^{man}\right]}^{*}$, produces the relative H- good at A at $p_{w}^{*}$, and its demand emerges at B, resulting in the managed country importing the fish resource-good.

On the other hand, the “open-access” country, whose autarky price is ${\left[p_{H}^{open}\right]}^{*}$, exports the H-good at $p_{w}^{*}$, which is given by the distance CB. This benefits the managed country because it increases the productivity of the resource-good sector. However, the higher world price stimulates the open access country to expand its more damaging sector's production, which leads to a decline in H-good productivity and lowers its consumption opportunities.

This result is also demonstrated by the positive correlation between welfare and $S_{\infty }\left(\beta^{*}\right)\text{,}$ as seen in the utility function. Thus, a managed country can rebuild its steady-state resource stock. By contrast, the open-access country experiences a decline in resource stocks in the post-trade steady-state equilibrium, resulting in the welfare changes discussed above.

### 3.2 Resource management by the domestic country

With only the domestic country's resource management, the results are different. Here, the manufacturing industry generates a more harmful resource-degrading impact than excessive resource extraction itself, denoted by Z>H(t).^17^ This creates an interesting point worth discussing, as a well-managed resource can lead to a shift in labor from a less harmful sector to a more depleting sector of fishery resources.^18^

**Fig. 2:**
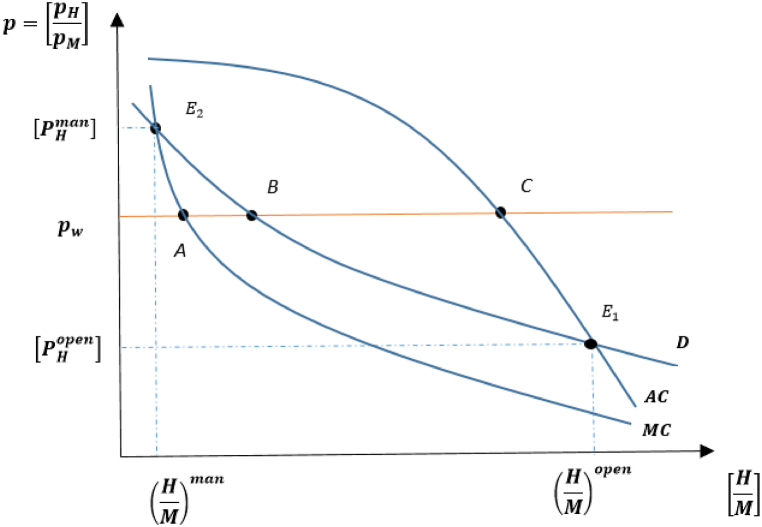
Open access and managed equilibria in autarky for domestic country.

Firstly, the model will specify the factors that determine the relationship between marginal cost (MC) and average cost (AC) in the domestic country, as illustrated in Fig. 2. Starting from the open-access case, labor shifts in the direction of the M-good under the managed case. Contrary to the previous case, the steady-state resource stock in the managed country is lower than that in the open-access country. Putting these results together (i.e., H(t)<Z and $S^{man}<S^{open}$) and Proposition 1 indicates that the resource good in the managed country is produced at lower levels than in the open access country, implying that the relative supply of fish harvest to manufacturing goods appears as follows: ${\left(\frac{H}{M}\right)}^{man}<{\left(\frac{H}{M}\right)}^{open}$. This figure verifies that the marginal cost curve in the domestic country is located further inside than the position of the relative average cost curve.

International trade benefits the open-access case, while worsening the welfare of the managed country. At the world price level, $p_{w}$, the open-access country exports the resource good and imports M- good from the managed country, as illustrated in Fig. 2 with points A, B, and C. This reflects the fact that the open-access country produces more of the harvest good, which is less environmentally harmful, thus improving post-trade steady-state resource stocks. This is the standard free trade result, as if a country operates its trade under competitive conditions, it will experience welfare-enhancing consequences by inducing labor to move to the more efficient fish harvesting sector.

However, the managed country's scenario is of particular interest because it indicates that in the absence of introducing appropriate regulation regimes for relatively more environmentally damaging economic activities (here, referring to the control and monitoring of the manufacturing sector), resource management might cause the expansion of the less efficient sector and the contraction of the more efficient activity, as observed in the managed country. This implies that the managed country can lose trade and government interventions in resource management can easily exacerbate the depletion problem in the related country.

These findings can be summarized as at least one country benefiting from trade. Furthermore, in the case of two identical countries, if only one implements a resource management regime, whether trade occurs between them will benefit the only managed country, depending on introducing the management regime to the appropriate harmful economic activity. The welfare analysis shows that in the case of a pessimistic scenario (regulating the resource sector when it is a relatively more environmentally friendly activity), trade can result in environmental deterioration and consequently harm the welfare of the managed country.^19^

## 4 International trade between different types of countries and resource management

In this section, the model considers two different types of “open-access” countries (domestic and foreign) that have trade relations with each other but in which only one country manages its fishery resource sector. The question here is whether this unilateral resource management policy can lead to welfare gains in both countries compared to the pre-management consequences, not to their closed economy results, but with the pre-management consequences. In other words, the present model focuses on the gainers and losers from this management regime implemented by only one of the two countries to regulate its fishery resource industry.

**Fig. 3:**
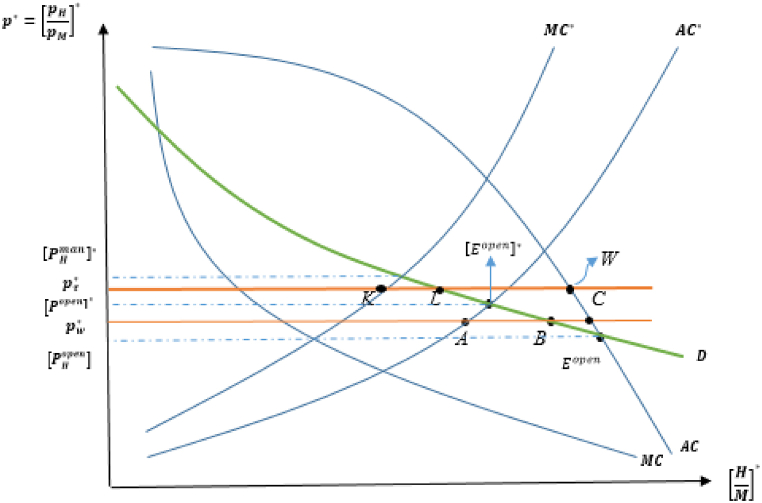
Open access (domestic) and managed (foreign) equilibria.

To investigate whether Pareto-improving unilateral resource management occurs, the model returns to the assumption that there is a difference in harvesting technology between trading partners, which motivates trade and determines trade and production patterns in the absence of resource management policies in either country. Remember that if $q<q^{*}$, then the total externality in the domestic country is lower than that of the foreign, $T<T^{*}$, resulting in that $S_{\infty }\left(\beta \right)>S_{\infty }\left(\beta^{*}\right)$ and ${p_{H}^{open}<\left[p_{H}^{open}\right]}^{*}$ if both countries are “open-access” countries. Here, the free trade price is $p_{w}^{*}$, which reflects the terms of trade $\left(p_{H}/p_{M}\right)$ at which the domestic country exports H-good (CB units) and the foreign country imports H-good (AB units), as depicted in Fig. 3.^20^

First, suppose that a foreign country manages its fishery resource industry, which increases the free trade relative price to $p_{\tau }^{*}$, positioned between the managed autarky price of the foreign in autarky, ${\left[p_{H}^{man}\right]}^{*}$, and the open-access price of the domestic in autarky, $p_{H}^{open}$. At the trading steady-state price $p_{\tau }^{*}$, both foreign (managed) and domestic (open-access) countries can gain, resulting in a win-win situation. An open-access (domestic) country has become better off because an increase in its exported goods induces the country to expand its fish resource industry, which is less harmful to fishery resources, thus improving efficiency in the fish harvest industry. Although an increase in the price of imported good ($p_{H})$ may worsen welfare to a certain extent, the managed (foreign) country will have less pressure on fishery resources by shifting its labor force to a more efficient sector (M-good) because KL=LM units of (H/M) are subject to trade (the domestic imports the fish resource good and the foreign imports it) at the free trade price, $p_{\tau }^{*}$. Consequently, the benefits of resource management can compensate for the loss associated with the negative impact of the terms of trade, implying that the managed country gains from trade.

**Fig. 4:**
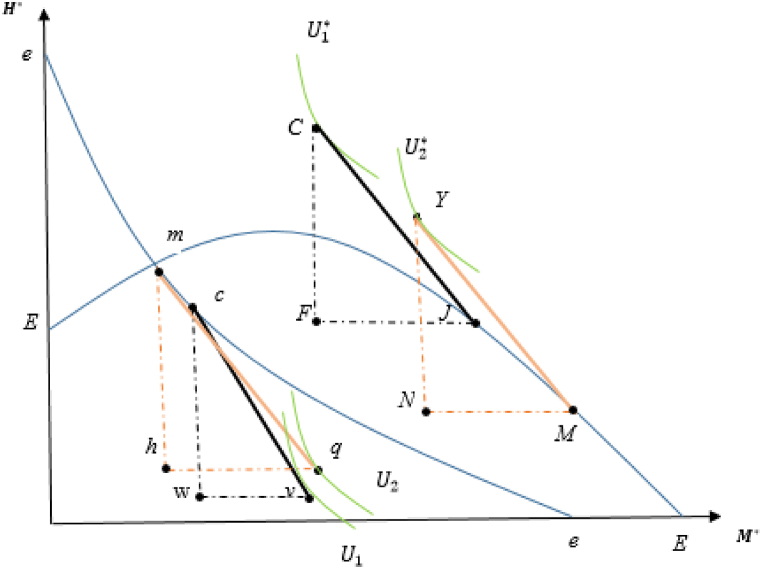
Open access (domestic) and managed (foreign) equilibria: Improving resource management.

This welfare analysis can be illustrated by iso-utility curves and production possibilities frontiers (PPFs) of both countries in Fig. 4, in which rr and RR represent the PPF curves for the domestic and foreign countries, respectively.^21^ Fig. 4 visualizes how welfare outcomes in each country change when the foreign country enforce resource management. This also provides an alternative way to determine welfare changes by using each country's utility function.

In the pre-management scenario, the free trade relative price is shown by the slopes of CJ and cq, in which the utility curves are represented by the iso-utility curves $U_{1}$ and $U_{1}^{*}$ for open access and managed countries, respectively. The discussion reveals that the relative price of the fish good increases when the foreign country manages its fishery resource sector, as indicated by the slopes of YM and cm. As fishery resource stocks recover in each country, both countries experience welfare-enhancing consequences for the above reasons.

This result is referred to as “improving resource management,” implying an intuition that when a country, in which the appropriate strategy is to regulate the resource sector to allocate more labor to a more efficient industry, manages its resource sector, trade can improve the economic well-being of both countries.^22^ This is a hopeful message from our findings, as it suggests that resource management in only one country may lead to post-trade welfare improvement and resource conservation.

The same consequences can be obtained using utility functions. Recall that $V^{open}=\Lambda +lnL+\beta \;\ln\left[qA\left(S_{\infty }\left(\beta \right)\right)\right]$ holds for an open-access (domestic) country. Since trade leads to labor allocation in favor of the resource industry, $\beta_{1}<\beta_{2}$ (denoting pre-management and post-trade labor allocation towards the resource sector, respectively), according to Lemma 1, $A\left(S_{\infty }\left(\beta_{1}\right)\right)<A\left(S_{\infty }\left(\beta_{2}\right)\right)$ is obtained, resulting in a positive welfare effect of trade in the open-access country.

Let us now focus on the utility function of a managed country. When the foreign country regulates its fishery resource industry, its real income consists of not only labor income ($L^{*})$, but also the resource rent ($p_{H}^{*}H_{S}^{*}-w^{*}L_{H}^{*}$) that relates to the fish harvest industry, which is assumed to be distributed equally among consumers in lump-sum form. Thus, income is given by $L^{*}+\left(p_{H}^{*}H_{S}^{*}-L_{H}^{*}\right)$. Substituting this expression into the utility function, we obtain the following equation, ${\left[V^{man}\right]}^{*}=\Lambda^{*}+\ln\left[p_{H}^{*}H_{S}^{*}-w^{*}L_{H}^{*}\right]+\beta^{*}\;\ln\left[qA\left(S_{\infty }\left(\beta^{*}\right)\right)\right]$, and then $A\left(S_{\infty }\left(\beta_{1}^{*}\right)\right)<A\left(S_{\infty }\left(\beta_{2}^{*}\right)\right)$ according to Lemma 1. Therefore, rebuilding the resource stock can enhance welfare terms in a managed (foreign) country based on the reasons explained above.

**Fig. 5:**
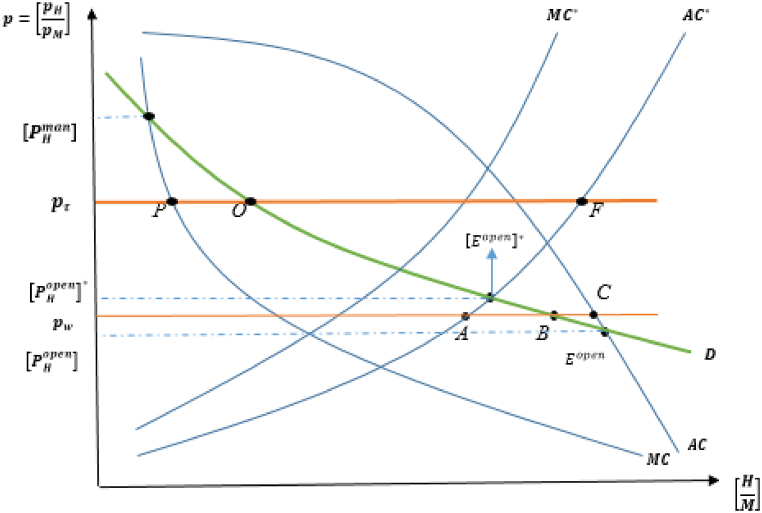
Open access (foreign) and managed (domestic) equilibria.

Having explained that it is possible for resource management enforced by the foreign country to improve welfare consequences in both countries, we will focus on the case in which the domestic country manages its resource sector and the foreign remains as an “open-access” country in the pre-management consideration. This situation is illustrated in Fig. 5, in which the relative price of free trade rises from $p_{w}$ to $p_{\tau }$, which lies between $p_{H}^{man}$ (the relative price of the resource good in the domestic (managed) country) and ${\left[p_{H}^{open}\right]}^{*}$ (the relative price of the resource good in the foreign (open access) country). Here, PO=OF units of (H/M) are traded. In this case, at $p_{w}$ in pre-management circumstances, the domestic country exports the H-good, and the foreign country exports the M-good, as in the previous case. However, this scenario highlights that the trade pattern is overturned when domestic resource management is enforced, in which the domestic becomes a net exporter of the M-good, and the foreign becomes a net exporter of the H-good.

When a domestic country manages its fish harvesting industry, the relative price of the resource good $\left(p_{\tau }\right)$ increases because of free trade. This higher relative price induces the open-access (foreign) country to export the resource good, leading to a depletion of fishery resource stocks from the pre-management level due to worsening open-access over-depletion, resulting in losses in productive efficiency and welfare. However, the managed (domestic) country shifts labor from a more productive and less harmful industry to a less productive and more harmful industry (M-exporter). This shift leads to higher industrial pollution, deteriorating resource stocks and reducing productivity in resource-intensive industries. As a result, welfare and resource conservation terms are diminished owing to the detrimental effect of the manufacturing good on fishery resource stock, which is a real lose-lose scenario.

**Fig. 6:**
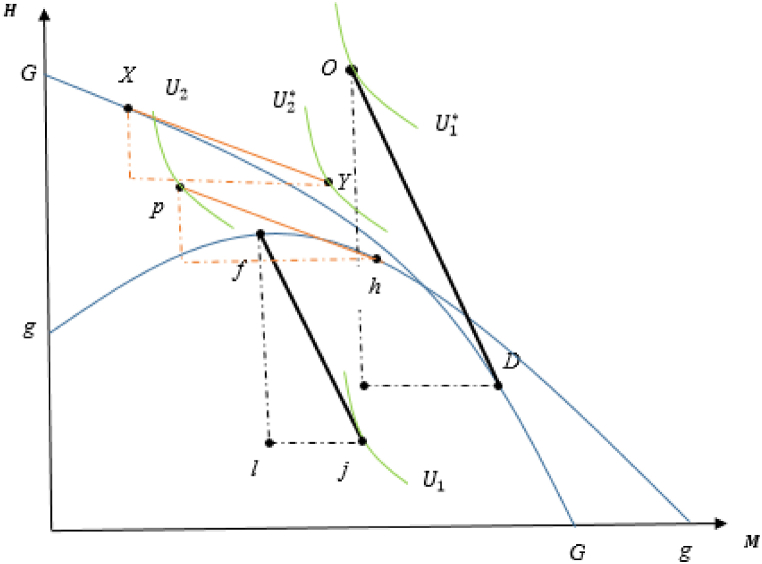
Open access (foreign) and managed (domestic) equilibria: Immiserizing resource management.

Following the previous situation, the analysis of welfare changes can be explained by the indifference curves of utility in each country, as visualized in Fig. 6, where gg and GG denote the PPF curves in domestic and foreign countries, respectively.

Remember that the indirect utility function in the open-access (foreign) country is ${\left[V^{open}\right]}^{*}=\Lambda^{*}+\ln\;L^{*}+\beta^{*}\;\ln\left[qA\left(S_{\infty }\left(\beta^{*}\right)\right)\right]$, and it is known that at trading price $p_{\tau }$, which is the slope of XY=ph, H expands and M contracts, leading to an increased over-depletion problem in the open-access economy. This leads to a depletion in fishery resource stocks, and opening up to trade tends to have welfare-decreasing consequences. For the managed country, this is $\left[V^{man}\right]=\Lambda +\ln\left[p_{H}H_{S}-wL_{H}\right]+\beta \;\ln\left[qA\left(S_{\infty }\left(\beta \right)\right)\right]$.

Here, the managed country's comparative advantage in M becomes stronger, and thus depleted fishery resource stocks diminish productivity in the environmentally sensitive sector. Considering the negative impact of an increase in the relative price of imported goods and depleted fishery resources, it is possible that the M exporter country can suffer welfare loss. This finding is related to Bhagwati's theory [42] of the immiserizing economic growth model.^23^ When the manufacturing sector in a managed country inflicts more harmful pressure on natural resources, free trade can exacerbate the depletion problem in resource conservation. Thus, it begets welfare-decreasing terms in both countries. The immiserizing resource management policy deserves a high degree of attention in the analysis.

A potential policy lesson for international organizations, such as the WTO, is that trade liberalization may occur as a welfare-reducing policy for all trading partners if resource management is enforced as a mandatory condition to be part of international trade. This is because such a resource management policy can change post-trade production patterns in favor of more damaging economic activity, reinforcing the deterioration problem in both countries. For this reason, this situation is the model's pessimistic scenario and is referred to as “immiserizing resource management.”^24^

Finally, this study focuses on how small-scale fisheries can be affected by these proposed resource management practices. It is worth mentioning that there are inadequate statistics for fishermen and other stakeholders aligned with small-scale fisheries, especially in developing South American and Southeast Asian countries [43,44]. These limitations require context-specific assessments to overcome overfishing and economic wastage [45]. In this model, resource management mainly and positively impacts the welfare of small-scale marine fisheries because they are much more dependent on fishery resources, especially in resource-dependent countries (Fig. 4). With an increase in the overall social welfare of the economy observed in both countries, when a resource-abundant country manages its open-access resources, small-scale fisheries may find themselves in a better post-trade situation because of the rebuilding of resource stocks. However, suppose resource management is imposed by a resource-scarce country, as shown in Fig. 6. In this case, small-scale fisheries are negatively affected by this management practice in the sense that long-term environmental degradation can lead to a decrease in the biomass of fishery stocks, as mentioned by Ref. [46].

## 5 Policy discussions and conclusion

This study analyzes the effects of trade liberalization and unilateral resource fishery management in countries with different technical standards. When a relatively more damaging pressure stems from resource extraction, a shift towards a more productive industry induced by unilateral resource management in the resource-good importing country can occur, resulting in welfare-improving consequences for both countries. That is, when the resource-good sector benefits from resource rent under fishery management, a resource-good importing country, whose fish harvest industry contracts are likely to benefit from trade. By contrast, trade will likely yield welfare gains in a resource-good exporting country by expanding the fishery sector.

On the other hand, a shift in the labor force towards a less-efficient industry owing to the management of the resource industry, which generates less resource-depleting activity in the resource-exporting country, worsens resource conservation. It decreases the welfare terms in both countries compared to the consequences of free trade. The key insight of this finding is simply that a well-managed unilateral resource may unintentionally yield “beggar-thy-neighbor’ and mutually harmful results, in which not only is the open-access trading partner worse off the expected consequences generated by the trade but also the managed country turns out to be disadvantaged. These results are in sharp contrast to those reported by Refs. [9,21], and [47]. This study includes two interconnected externalities that change the nature of the production possibilities frontier, production patterns, and post-trade welfare terms, which sets it apart from the aforementioned studies. This study contributes to the literature on trade liberalization and unilateral resource management.

These findings emphasize the importance of policy-relevant interventions when resource management is a prerequisite for participation in international trade. Depending on the findings that these consequences are situation-specific, this study suggests there may not be a certain case in which unilateral resource management will always provide a relative advantage in international trade. Therefore, the model shows that the efficiency of unilateral fishery management policies in improving well-being and conservation depends fundamentally on whether resource extraction is a major source of environmental deterioration. It seems that opening up trade may not always be politically correct, and thus unnecessary persistence of trade openness and resource management may bring about undesirable outcomes. Moreover, this finding implies that complete fishery management in an open-access harvesting industry is not a requirement to obtain welfare-enhancing consequences from trade if trade does not exacerbate the over-exploitation of open-access resources. Another important policy implication of this study is that policymakers in developing countries should be concerned with which externalities are more detrimental to resource conservation, and which economic activities should be managed first. These results differ from most of the findings in the literature in which resource management benefits one or both countries under open-access conditions.

In practice, a potential environmental policy lesson for a managed resource-scarce country is that industrializing at the expense of contracting environmentally sensitive sectors can yield welfare losses because resource management strategies can lead to more distorting industries to expand. In such a framework, well-defined and classified distortions and institutional failures turn out to be more important for policymakers to reach appropriate ecological targets. Another practical policy recommendation is that, particularly in developing countries with a comparative advantage on pollution-generating products using resource goods as an intermediate input factor, polluter firms should eagerly cooperate with lawful authorities to regulate negative externalities that could be derived from producing manufactured goods. The reason is that the regulation of the inappropriate sector, which elicits environmental deterioration, slows down the growth rate of these industries in the long term [48]. Note that generalizations can appear oversimplified, and an empirical study is always necessary to measure the policy suggestions proposed in this study.

The present model develops a theoretical setting for an open-access resource economy to conduct the analysis. In line with the analytical literature on resource extraction [[9], [10], [11],[49], [50], [51]], this paper also integrates the industrial externality problem of the manufacturing industry, which creates different types of countries with opposite production-environment relations. The dominance of externalities over each other matters because this attribute determines post-trade production patterns, welfare consequences, and resource protection. The model considers unilateral resource management enforced by different types of country in each scenario. Let the domestic country serve as a proxy for resource-scarce countries in which unilateral resource management is not an optimal or effective policy. The foreign, however, represents resource-abundant countries where fishery management is a beneficial strategy for yielding welfare-improving results. This theoretical construction allows us to examine the trade-resource management connection in a richer and more realistic framework. To the best of my knowledge, this formal analysis of such different scenarios has not been considered in the context of trade between emerging countries.

The strength of the model lies in its ability to incorporate the more realistic characteristics of detrimental externalities and cross-country heterogeneity when determining optimal resource management decisions under different scenarios. Through this approach, the model brings to light an often-overlooked point: If resource management is not designed correctly, meaning that the economy unintentionally manages its resource sector, which is a less resource-depleting activity that instead of monitoring more harmful sectors, it may lead to an expansion of more harmful economic activity in both countries, resulting in immiserizing resource management as for the welfare effects of trade liberalization. This result may clarify why for some fisheries, there is an unwillingness to enforce full fishery resource management strategies, particularly in developing countries. This analysis suggests that contrary to conventional wisdom, resource management may be counterproductive under certain conditions.

I am aware that these findings rely on theoretical assumptions that have enabled me to construct a highly preliminary model. The particular functional forms of resource stock, production, and consumption restrict the validity of the results in general cases. A potentially interesting extension of this study is the determination of a model's resource management policy. Endogenizing the choice of management strategy enables us to consider different levels of management standards. Furthermore, it would be interesting to implement a multilateral resource management approach for the model in this study.

## Statements and declarations

### Author contribution statement

GÖKHAN GÜVEN: Conceived and designed the experiments; Performed the experiments; Analyzed and interpreted the data; Contributed reagents, materials, analysis tools or data; Wrote the paper.

### Data availability statement

No data was used for the research described in the article.

## Funding statement

This research received no specific grant from any funding agency in the public, commercial, or not-for-profit sectors.

## Declaration of competing interest

The authors declare that they have no known competing financial interests or personal relationships that could have appeared to influence the work reported in this paper.

## Footnotes

1 This does not mean enforcing individual transferable quotas, regulations, tax policies, or other private control regimes does not solve unwarranted extraction problems. This study attempts to choose the best alternative solution to these externalities while avoiding political considerations and highly resource-costly solutions such as applying resource management regimes.

2 Resource management cooperation can solve distortions pertaining to open-access assumptions when using resources. However, it is challenging to obtain cooperative solution sets because trading partners generally have different levels of economic payoffs, implying that enforcing binding contracts is more challenging [52,53]. That is, it is considerably difficult to manage fishery stocks cooperatively, implying that non-cooperative and one-sided management strategies are dominant [54]. [55] provided evidence about the consequences of collective action to protect forestry in Nepal and concluded that efficient outcomes could be achieved with cooperation, but conflicts between countries would make cooperation eminently challenging. Therefore, this model focuses on unilateral resource-management strategies.

3 To distinguish the domestic country's variables from those of the foreign country, the model includes asterisks to identify the variables of the foreign country.

4 Renewable resource stock can be considered a proxy variable that measures the Environmental Performance Index formulated by Yale and Columbia Universities. Please, I refer the reader to following website https://epi.yale.edu/epi-results/2022/component/epi.

5 The pollution intensity parameter can be considered a non-negative parameter that characterizes the difference between the actual state of technical knowledge and technological abatement [56]. In general, as in the present model, pollution is measured as a function of the production process [57]. For stricter standards of pollution intensity parameters, industrial firms are expected to produce less than optimal in a perfectly competitive market [58]. Moreover [59], showed that tight regulatory policies on pollution-producing firms might cause offshoring of the urban manufacturing sector to countries with laxer standards. For empirical studies on the relationship between pollution intensity parameters and international trade, I refer the readers to [60,61].

6 This definition provides a threshold between resource abundance and resource-scarce countries. As discussed by [62], Tunisia and Morocco are typical examples of resource-abundant economies. Moreover [63], demonstrated that China and India are standard examples of resource-scarce countries with lower resource productivity and a higher number of polluter firms [[104], [105], [20]].

7 Overharvesting and pollution generate problems in resource stocks. Both environmental burdens are considered to be major by-products of economic activities that simultaneously deteriorate fishery or marine species. These twin pressures are identified as intra-industry (open-access) and inter-industry (pollution) detrimental stock impacts, which create complexities in determining the evolutionary path of natural resource stocks. The combination of excessive harvesting and industrial pollution may lead to the complete extinction of natural resources [64]. Such a depletion was observed in tree species in the Sudetes area in 1995. Additionally [65], focused on leeches, which are endangered, and concluded that the excessive use of fertilizers and pesticides as chemical agents and overutilization of these animals synergistically increases the risk of extinction.

8 This model predicts that enhancements in harvesting technology indicate a better ability to restrict resource harvesting and contribute to property-rights-based resource management regimes [66,29]. [67] noted that this relationship is reciprocal, meaning that different rights-based management practices would also influence advancement in the technological progress of harvesting. Improvements in harvesting technology can also encourage the overexploitation of renewable resources, which may cause extinction [68].

9 This also implies that H(t)<Z holds in the domestic country and Z<H(t) in the foreign country in terms of their respective depleting impacts on fishery resources. The sensitivity of the relative magnitude of externality parameters on the relationship between trade liberalization and environmental degradation is empirically examined in different models such as [[69], [70], [71]]. [69] showed that when resource stocks are exposed to open-access problems, the trade openness deteriorates the forest recovery rates and leads to deforestation in non-OECD countries. However, it decelerates the degradation problem in OECD countries [70]. found that intra-industry externality on environmental quality (denoting pollution problem in this model) in the case of complete trade openness is identified as less pro-environment of 200 countries from 2000 to 2005 [71]. provided empirical evidence that environmental pollution was exacerbated by international trade rather than intra-industry trade alternatives, negatively impacting agricultural land quality in European Union countries from to 2000–2014.

10 This Lemma is consistent with the empirical results. Tajikistan is highly dependent on natural resource export revenue in its trade volume [72]. explores how labor migration from Tajikistan to the Russian Federation impacts natural resource preservation and concludes that population movement ensures a significant enhancement in environmental quality [73]. also constructed a model in which they examined the effects of hiring labor by the industry sector from the local agricultural sector and found that as industrialization gets more and more advanced, which increases the possibility of experiencing more relative dominance of manufacturing activity, the migration of unskilled worker to urban boosts up the environmental deterioration [74]. studied the relationship between rural migration and the rural environment in Southwest China. This study empirically evaluates what criteria can facilitate an understanding of how the environmentally sensitive sector to polluter-producing industry migration impacts resource preservation. Consequently, he found that labor migrants could degrade environmental conservation compared to non-labor migrants.

11 The model assumes that the price of the manufacturing good is taken as numeraire, and then the relative price of the harvesting good is denoted by $p=p_{H}/p_{M}=p_{H}$.

12 The utility function in the foreign country can be expressed as ${V^{*}}^{\left(open\right)}=\Lambda^{*}+lnL+\beta^{*}\;\ln\left[q^{*}A\left(S_{\infty }\left(\beta^{*}\right)\right)\right]$.

13 Resource management often involves limiting the amount of resource extraction or use, establishing protected areas or reserves, and promoting sustainable practices, such as reforestation or sustainable fishing. On the other hand, pollution control focuses on preventing, minimizing, or mitigating the release of pollutants into the environment. This could involve regulating factory emissions, controlling farm runoff, or managing waste disposal. Pollution control measures may also include monitoring and enforcement to ensure that the environmental standards are met.

14 [75] demonstrated that the best practice to identify resource management in resource–abundant countries (Norway, Chile, and Canada) is to implement and collect resource rents, which is the policy that the present model identifies due to resource management practices.

15 When fish harvest is carried out under open-access conditions, the relative supply curve equals the relative average cost. However, when a resource good is produced under managed conditions, the marginal cost curve represents the relative supply curve for both countries.

16 The model assumes that the fishery resource will be managed under the zero-discount rate assumption, which means that the associated resource rent will be maximized in the long run. The managed situation implies that the steady-state fishery resources, satisfying dS/dt=0, and taking $p_{H}$ as the price of the fishery good, must equal the marginal cost of fish harvest. This increases the real income of the managed country, which is distributed to consumers in the economy using the lump-sum method. In other words, the open-access country's real income is defined as wL=L, as presented in the utility function. In the managed country, however, income is $\left[wL_{M}+p_{H}H_{S}\right]\text{,}$ which is the total wage income and additional resource rent arising from resource extraction.

17 In this context, the domestic country is considered resource-scarce [76]. investigated the impacts of natural resource management (in the form of environmental taxes or green investment) and concluded that such policies abate environmental emissions, particularly in China.

18 Especially in industrialized countries, there are concerns about the decline in environmental quality, resource protection, and deterioration of the ecological environment, which claim that industrial pollution is the main force behind these detrimental results [77]. Therefore, it has been widely confirmed that strict regulations of polluting firms improve natural resource stocks and reduce environmental pollution problems [78]. Although many developed countries have begun introducing important pollution control programs to mitigate associated problems, many still do not take the necessary steps because the estimated costs for introducing pollution control programs may exceed the benefits [[79], [80], [81]]. Consequently, resource management can come to the fore, even if it does not solve the primary depleting activity problem.

19 This result contrasts with the findings of [9,82], and [21], which all claim that under conditions of well-defined property rights, both trading partners gain from trade. However, the present model adds a new requirement to their results and states that it is not only sufficient to properly define property rights, but it is also important to eliminate the problem of property rights in the relatively and environmentally more harmful industry to prevent labor from migrating to the less efficient sector.

20 AB=CB equation must hold at the $p_{w}^{*}\text{.}$

21 It is known in the existing literature that when excessive harvesting is more detrimental to resources than industrial pollution, the production possibilities frontier is convex-shaped. On the other hand, when industrial pollution is more resource-depleting than resource extraction, the production possibilities frontier becomes concave-shaped in the M − H commodity space (see for a detailed discussion: [18,20,83]. Because this result is valid in this model with the same logic, it seems unnecessary to derive the PPF curves again.

22 Cooperation in the management of resources is desirable but not mandatory because non-cooperative resource management (in this context, it is represented by unilateral resource practices) is formally acceptable, even if resource stocks are shared internationally [84,85]. This unilateral policy resembles the self-interest model, in which economies seek to maximize their welfare without focusing on collective action [86]. Restricting only one country's access to renewable resource stocks may be sufficient for sustainability, resource preservation, and welfare gains in the long run. Especially for resources on the brink of extinction, an individualistic strategy can be sufficient to moderate overexploitation resource use [87]. The results in this subsection are compatible with [88]'s and [89] claims that controlling and monitoring resource stocks should prioritize intranational interests.

23 Economic growth and environmental preservation cannot be maximized concurrently [90]. The general proposition is that economic development with urbanization can generate an acceptable amount of pollution and a deteriorating environment in the early stages of growth [91]. [92] proposed that challenges caused by economic expansion, particularly in the short term, could readily lead to the degradation of natural resources and sometimes unforeseen implications for the environment, consequently resulting in unsustainable growth in the long term (see Ref. [93]) for an empirical analysis focusing on the negative impact of growth on environmental performance). This discussion necessitates environmental regulations, enforcing tighter resource management policies, and new institutions to conserve environmental quality and decrease the ecological footprint in the path of economic boosts [94,95]. Trade liberalization also plays a vital role in increasing products with higher carbon levels [96]. Therefore, international trade reallocates manufacturing industries to developing countries that are less environmentally regulated [97]. To promote sustainable resource management, international trade organizations can establish regulatory rules that trading countries must follow to be part of international trade. The rules for maintaining a sustainable management path can be the form of preserving soil and water quality, landscape-scale techniques, and the control of pest plants and animals [98]. Finally, a more recent study by Ref. [99] proposed that trade openness can cause countries to shift from preservation to extinction. In return, depleted natural resources have reduced economic growth.

24 In non-cooperative management decisions, each agent makes a management and control action without considering its influence on the other partner's welfare terms [100]. Immiserizing resource management is a typical example of an unintended consequence of non-cooperative resource management that can occur in the high seas [101,102]. [101] empirically determined that the average resource stock biomass in the non-cooperative case is lower than that in cooperative actions, as shown using a particular example of the cod fishery in the Northeast Atlantic (see Ref. [101], p. 446) [103]. found an empirical result that stock size is reduced when one-sided resource management practice is implemented compared to joint management strategy, consequently resulting in welfare loss, as shown in the present model [95, p. 55]. This result is also called ‘black growth’ [85].
